# Tissue Compartment Analysis for Biomarker Discovery by Gene Expression Profiling

**DOI:** 10.1371/journal.pone.0007779

**Published:** 2009-11-10

**Authors:** Antoine Disset, Lydie Cheval, Olga Soutourina, Jean-Paul Duong Van Huyen, Guorong Li, Christian Genin, Jacques Tostain, Alexandre Loupy, Alain Doucet, Rabary Rajerison

**Affiliations:** 1 UPMC Univ Paris 06, UMRS 872, Laboratoire de génomique, physiologie et physiopathologie rénales, Paris, France; 2 CNRS, ERL 7226, Laboratoire de physiologie et génomique rénales, Paris, France; 3 Institut Pasteur, URA CNRS 2171, Unité de génétique des génomes bactériens, Paris, France; 4 Service d'Urologie-Andrologie, CHU, Saint Etienne, France; 5 Laboratoire d'Immunologie Clinique, CHU, Saint Etienne, France; Dr. Margarete Fischer-Bosch Institute of Clinical Pharmacology, Germany

## Abstract

**Background:**

Although high throughput technologies for gene profiling are reliable tools, sample/tissue heterogeneity limits their outcomes when applied to identify molecular markers. Indeed, inter-sample differences in cell composition contribute to scatter the data, preventing detection of small but relevant changes in gene expression level. To date, attempts to circumvent this difficulty were based on isolation of the different cell structures constituting biological samples. As an alternate approach, we developed a tissue compartment analysis (TCA) method to assess the cell composition of tissue samples, and applied it to standardize data and to identify biomarkers.

**Methodology/Principal Findings:**

TCA is based on the comparison of mRNA expression levels of specific markers of the different constitutive structures in pure isolated structures, on the one hand, and in the whole sample on the other. TCA method was here developed with human kidney samples, as an example of highly heterogeneous organ. It was validated by comparison of the data with those obtained by histo-morphometry. TCA demonstrated the extreme variety of composition of kidney samples, with abundance of specific structures varying from 5 to 95% of the whole sample. TCA permitted to accurately standardize gene expression level amongst >100 kidney biopsies, and to identify otherwise imperceptible molecular disease markers.

**Conclusions/Significance:**

Because TCA does not require specific preparation of sample, it can be applied to all existing tissue or cDNA libraries or to published data sets, inasmuch specific operational compartments markers are available. In human, where the small size of tissue samples collected in clinical practice accounts for high structural diversity, TCA is well suited for the identification of molecular markers of diseases, and the follow up of identified markers in single patients for diagnosis/prognosis and evaluation of therapy efficiency. In laboratory animals, TCA will interestingly be applied to central nervous system where tissue heterogeneity is a limiting factor.

## Introduction

A central goal in biomedicine is to identify specific markers for diagnosis and prognosis of diseases and for evaluating treatment efficiency. Identification of molecular biomarkers is often based on differential profiling of gene expression [1], [2]. Although powerful technologies for gene expression analysis, e.g. microarrays and SAGE [3], are nowadays well systematized and highly reliable, the overall procedure for differential gene expression profiling still suffers from several flaws. One seldom solved relates to the very nature of the biological samples, especially when studying heterogeneous tissues or organs [4], [5]. As a matter of fact, random sampling of a heterogeneous tissue yields samples with different cell compositions. Thus, differences in gene expression levels observed between samples may be accounted for not only by true changes in gene expression, but also by differences in their cell composition. This artefact increases data scatter and may prevent detection of small amplitude changes in gene expression, as those expected for early biomarkers.

Because the diversity of tissue samples composition is inversely related to their size, this pitfall could theoretically be circumvented by analyzing tissue fragments large enough to be representative of the average composition of the whole tissue. Unfortunately, most often this is not feasible for human tissues/organs since, for obvious reasons, tissue biopsies are downsized to the minimum required for histoimmunopathological analysis. Two types of human biological material are not subject to this difficulty: the blood, because fairly large volumes are readily available which allows separating the different cell populations, and tumors because they mainly consist of a clonal mass of tissue. This likely explains that differential gene expression profiling has led to important achievements in hematology and oncology [6], [7], whereas outcomes remain disappointing in other medical fields.

Laser capture micro-dissection (LCM) can provide pure preparations of the different structures from heterogeneous organs or tissues [8]–[10]. However, LCM remains tedious, especially when coupled with procedures for high quality RNA extraction, and is difficult to set up for routine use in clinical laboratories. Alternately, we developed a tissue compartment analysis (TCA) method that allows quantifying the fractional volume of the different structures constituting tissue samples and solving the problem of tissue sample heterogeneity, and applied it to identify biomarkers.

## Results

### Principle of the Method

Calculation of the fractional volumes of the different constitutive structures of any sample is based on the comparison between mRNA expression levels of specific markers of the different constitutive structures in pure isolated structures, on the one hand, and in the whole sample on the other. The fractional volume of any structure X (%V_x_) is given by:
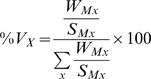
where W_Mx_ and S_Mx_ designates the abundance of a X-specific transcript marker (Mx), in the whole sample and in the pure structure X respectively.

This TCA method was applied to human kidney, as an example of highly heterogeneous tissue, using both normal and pathological kidney tissue. The analysis was restricted to the four main structures that constitute human kidney biopsies, i.e. glomeruli (G), proximal convoluted tubules (PCT), cortical thick ascending limbs of Henle's loops (cTAL) and cortical collecting duct (CCD). W_Mx_ was quantified by RT-PCR and, for S_Mx_, we took advantage of published data from SAGE libraries generated from pure populations of the different structures constituting human normal kidney tissue [11] (**table 1**).

**Table 1:** Occurrence in glomerular and tubular SAGE libraries from human kidneys of the specific tags of genes analyzed in this study.

	G	PCT	cTAL	CCD	Tag sequence
**Structure-specific markers**					
PODXL, *Podocalyxin-like (NM_001018111)*	129	0	0	0	ATATATGTCT
WT1, *Wilms tumor 1 (NM_000378)*	18	0	0	0	TTACAAGATA
ALDOB, *Aldolase B (NM_000035)*	0	307	7	2	AAATTTCACA
SLC13A3, *Solute carrier family 13 member 3 (NM_022829)*	0	71	0	0	TGGGGTCTGT
SLC12A1, *Na/K/2Cl cotransporter 2 (NM_000338)*	0	0	149	1	TGAGCAATCA
UMOD, *Uromodulin (NM_003361)*	3	3	837	4	AATCCCGTGT
AQP2, *Aquaporin 2 (NM_000486)*	0	1	3	157	ACACACACCA
FXYD4, *Corticosteroid hormone induced factor (NM_173160)*	0	2	0	191	AGGAGGCTTC
**Ubiquitous reference genes**					
RPLP1, *Ribosomal phosphoprotein, large, P1 (NM_001003)*	53	49	59	74	AATGCCCTCA
RPL19, *Ribosomal protein L19 (NM_000981)*	124	98	75	83	AGCCATTAAA
PPIA, *Peptidylpropyl isomerase A (NM_021130)*	8	12	17	20	ATTTGGTGTG
**Genes selected for data standardization**					
DUSP9, *Dual-specificity phosphatase 9 (NM_001395)*	0	1	30	0	TATGCTTGTT
GSTA1, *Glutathione S-transferase α1 (NM_145740)*	1	43	4	0	TGATGTGAAT
KCNJ1, *Renal outer-medullary potassium channel (NM_153767)*	0	3	27	23	CCCACCTGCA
MUC1, *Mucin 1 (NM_002456)*	1	2	21	26	CTGAACTGGA
NPHS2, *Podocin (NM_014625)*	68	0	0	0	CCTCACTGAA
PTGER1, *Prostaglandin E receptor 1 (NM_000955)*	0	0	0	21	CTGGACCCTT
**Pathology biomarkers**					
SERPINH1, *Serpin peptidase inhibitor, H1 (NM_001235)*	1	1	0	2	AAGCCTGCCT
COL4A5, *Collagen, type IV, alpha-5 (NM_000495)*	2	0	0	0	AAGATAACAT
CTGF, *Connective tissue growth factor (NM_001901)*	239	2	4	5	AGTTTTTTCA
DCN, *Decorin (NM_001920)*	107	0	0	0	AAGTGACTTC
TRPC6, *Transient receptor potential cation channel C6 (NM_004621)*	1	0	0	0	AGAAAATACA

Data, from Chabardès-Garonne *et al* [11]. correspond to tag counts normalized to 50,000 total tags per library. Human Genome Organization (HUGO) gene symbol is followed by a usual name and the RefSeq identification. SAGE data are available at GEO (www.ncbi.nlm.nih.gov/geo/) (GSM10419, GSM10423, GSM10426 and GSM10428). Tag sequences are also provided.

### Specific Markers of Human Kidney Structures for TCA

**Figure 1:**
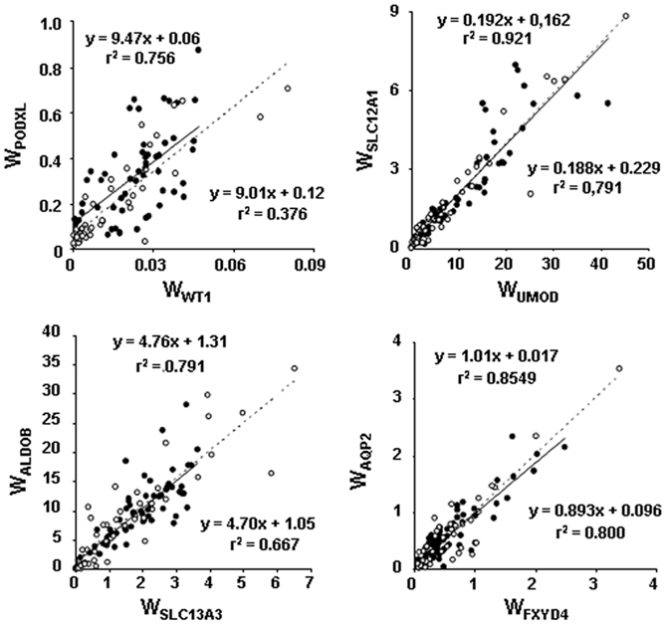
Expression of structure-specific markers in human kidney. Relationship between the expression levels of specific markers of glomerulus (PODXL and WT1), proximal tubule (ALDOB and SLC13A3), cortical thick ascending limb of Henle's loop (SLC12A1 and UMOD) and CCD (AQP2 and FXYD4) in >60 normal (closed symbols) and >40 pathological kidney samples (open symbols). For each structure, the slopes of the regression lines corresponding to normal (full line, equation and R^2^ at bottom right) and pathological samples (dotted line, equation and R^2^ value at top left) were not statistically different (t test).

To minimize quantification errors, two transcript markers were utilized for each structure. The four couples of markers were selected among candidates in human tubule SAGE libraries [11] based upon the following criteria: 1) they are expressed at high level to facilitate accurate PCR quantification (**table 1**); 2) they encode proteins relevant to specific functions of the different kidney structures, so that their expression is likely negligible in non considered kidney structures, and 3) their expression should display minimal inter-individual differences. Based on these criteria, we selected podocalyxin-like (PODXL) and Wilms tumor 1 (WT1) for glomerulus, aldolase B (ALDOB) and the solute carrier SLC13A3 for proximal tubules, the Na/K/2Cl co-transporter NKCC2 (SLC12A1) and uromodulin (UMOD) for thick ascending limbs of Henle's loop, and aquaporin 2 (AQP2) and CHIF (FXYD4) for CCD. Data from the literature and from SAGE libraries (Table 1) indicate that these transcripts are specific markers of the different kidney structures, and results in figure 1 (filled circles) show the correlations existing between the expression levels determined by RT-PCR of these four pairs of structure-specific markers in >60 normal kidney samples from different patients. For PCT, cTAL and CCD markers, a high correlation (0.66<R^2^<0.80) existed between expression levels of the two markers whereas the correlation was weaker for the glomerulus markers (R^2^ = 0.38). Importantly, the slope of the regression line (the ratio of the W_M_ of the two markers) was close to the calculated ratio of occurrence of the cognate tags in SAGE libraries: glomerulus, W_PODXL_/W_WT1_ = 9.0, S_PODXL_/S_WT1_ = 7.2; PCT, W_ALDOB_/W_SLC13A3_ = 4.7, S_ALDOB_/S_SLC13A3_ = 4.3; cTAL, W_SLC12A1_/W_UMOD_ = 0.19, S_SLC12A1_/S_UMOD_ = 0.18; CCD, W_AQP2_/W_FXYD4_ = 0.89, S_AQP2_/S_FXYD4_ = 0.82. This demonstrates the quantitative equivalence of SAGE and RT-PCT data. The selected markers proved to be usable also for pathological tissue (**Fig. 1** **, open circles**) since a high correlation (0.76<R^2^<0.92) existed between expression levels of the two markers for the four structures in >40 pathological kidney samples, and the slope of the regression lines for normal and pathological samples were not statistically different (0.1<t<1.3).

### Validation of TCA Method

**Figure 2:**
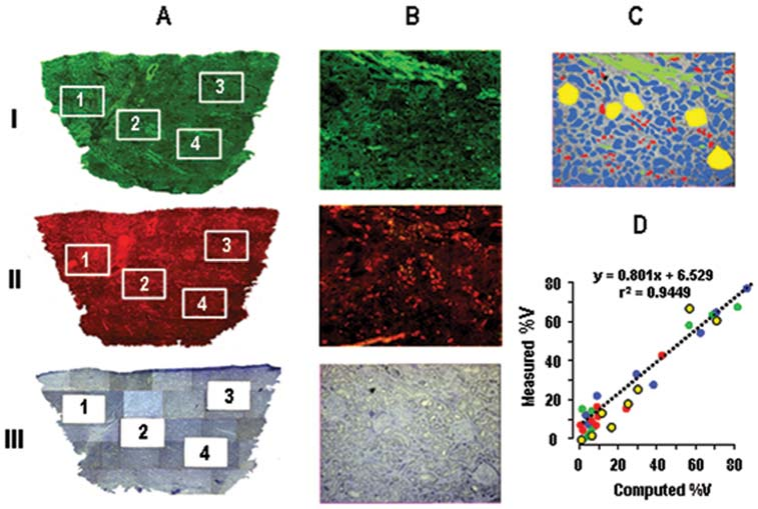
Validation of TCA by histo-morphometric analysis of kidney tissue composition. **A**. Overview of three kidney serial sections stained with anti-uromodulin antibody (I), anti-AQP2 antibody (II), and toluidine blue (III, micrographs after LCM). The zones (1 to 4) used for analysis are delineated. **B**. Higher magnification images of zones 4 (before LCM for section III). **C**. Image analysis of zone 4 allowing the construction of the color-coded image (G, yellow; PCTs, blue; cTAL, green; CCDs, red; grey, remaining tissue) used for determination of the surface area of the different compartments. **D**. Relationship between TCA-computed and measured fractional volumes of the 4 compartments in 8 samples analyzed as described in C (same color code as in C). The slope of the regression line was not statistically different from 1 (t test).

TCA method was validated by comparing the sample composition calculated as indicated above with that provided by histo-morphometric analysis on serial normal kidney sections adjacent to that used for RT-PCR quantification of structure markers (**Fig. 2A–2C**). Results obtained on 8 LCM-derived tissue pieces from two different kidneys (**Fig. 2D**) demonstrated a strong correlation (R^2^ = 0.945) between the fractional volume of the different kidney compartments determined by TCA method and by histo-morphometry in all samples analyzed. In addition, the slope of the regression line was not statistically different from 1 (t = 1.03). This not only confirms the accuracy of the TCA method, but also that of the underlying pre-requisite, i.e. the comparability of SAGE and RT-PCR data.

### Diversity of Human Kidney Samples

**Figure 3:**
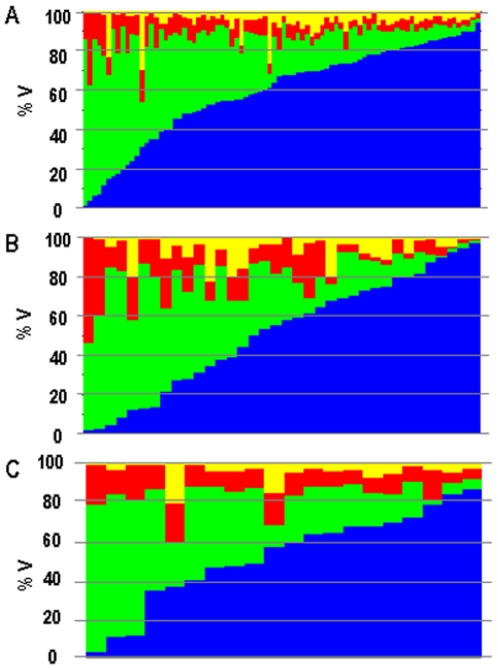
Structural heterogeneity of samples. TCA-computed fractional volumes of proximal convoluted tubules (PCT, blue), glomeruli (G, yellow), cortical thick ascending limbs of Henle's loop (cTAL, green) and aldosterone-sensitive distal nephron (CCD, red) in 94 normal samples (A), 36 pathological needle biopsies (B) and twenty fragments of normal tissue from a same patient (C). Samples are ranged according to increasing fractional volume of PCTs.

TCA revealed a wide spectrum of representations of the different structures among >90 normal samples (**Fig. 3A**) and >30 pathological samples (**Fig. 3B**) from different patients, e.g. the fraction constituted by PCTs varies from 1 to >95% of the whole sample. This diversity is related to tissue sampling rather than to inter-patient variations since a similar diversity was observed between twenty fragments from a same kidney (**Fig. 3C**).

### Data Standardization

These marked differences in tissue composition constitute a major determinant of inter-sample variance in gene expression level for all genes unevenly expressed in the different structures. This can be overcome by standardizing data with a gene- and sample-specific factor (SF) which accounts for both the differential fractional volume of and gene expression in the various structures
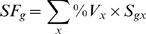
where S_gx_ is the abundance of the transcript (g) in the different compartments (here, the occurrence of the candidate transcript tag).

**Figure 4:**
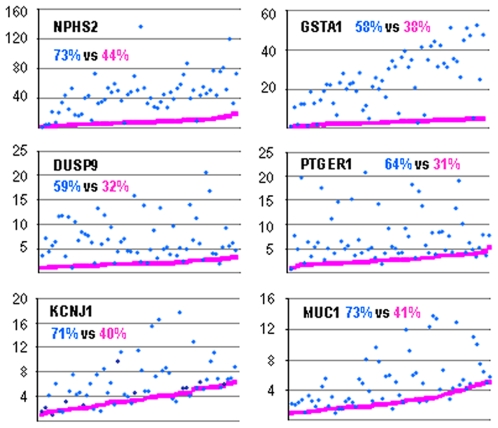
Data standardization. Expression of NPHS2 (podocin), GSTA1 (Glutathione S-transferase α1), DUSP9 (Dual-specificity phosphatase 9), PTGER1 (Prostaglandin E receptor 1), KCNJ1 (Renal outer medullary potassium channel, ROMK1) and MUC1 (Mucin 1) in 60 normal samples was standardized either by the reference gene RPLP1 (blue points) or by SF (pink points). Similar results were obtained when using RPL19 or PPIA as reference genes. Data are expressed as fold of the lowest value, and samples are ranged according to increasing SF-standardized values. Values in the graphs are the variances for RPL1-normalized (blue) and SF-normalized data (pink).

When compared to classical data standardization procedure using RPLP1 as a so-called reference gene[12], standardization with SF markedly reduced data scatter as the variance was reduced 1.6–2.0 fold (**Fig. 4**).

### Identification of Pathology Markers

**Figure 5:**
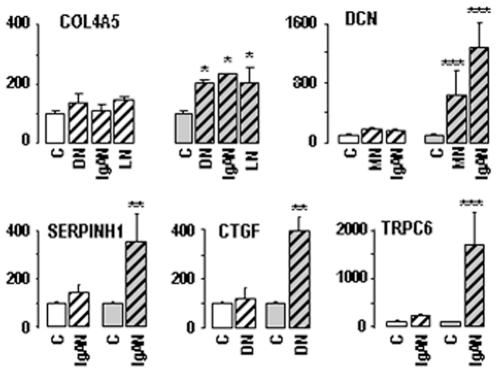
Identification of pathology markers. Expression of COL4A5 (collagen type IV, alpha-5), DCN (decorin), SERPINH1 (serpin peptidase inhibitor, H1), CTGF (connective tissue growth factor) and TRPC6 (transient receptor potential cation channel C6) in normal tissue (open columns C, n = 64) and pathological biopsies (hatched columns): DN, diabetic nephropathy (n = 7); MN, membranous nephropathy (n = 7); IgAN, IgA nephropathy (n = 5); LN, lupus nephropathy (n = 7). Data were standardized using either the reference gene RPLP1 (left, open columns) or SF (right, grey columns), and results are expressed as percent ± SE of normal group. Statistical comparisons with normal groups were performed by one way ANOVA followed by Holm-Sidak test: *, p<0.01; **, p<0.005; ***, p<0.001.

Using immuno-histochemistry or *in situ* hybridization, i.e. analytical methods that palliate kidney heterogeneity, authors reported increased expression of putative markers in several kidney pathologies [13]–[17]. We therefore searched whether standardization of data would allow identification of these markers by RT-PCR on heterogeneous kidney samples. Results in **Fig. 5** show that, conversely to classical standardization procedure, standardization with SF allowed revealing the over-expression of such pathological markers.

### Localization of Gene Expression

**Figure 6:**
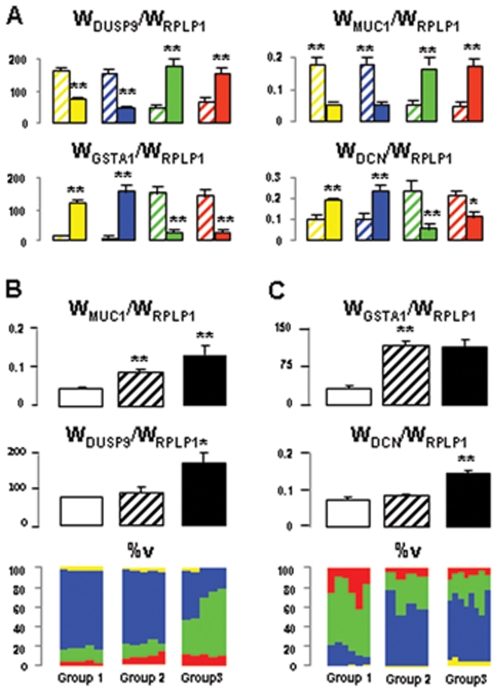
Localization of gene expression. Determination of the composition of kidney samples may serve to localize gene expression along the nephron through comparison of expression levels in subgroups of samples with different enrichment in a given structure. This is illustrated for DUSP9 (Dual-specificity phosphatase 9), MUC1 (Mucin 1), GSTA1 (Glutathione S-transferase α1) and DCN (decorin), by comparing (**A**) eight subgroups made of the ten samples with the lowest (hatched columns) and the highest (full columns) proportions of G (yellow), PCT (blue), cTAL (green) and CCD (red), (**B**) subgroups with similar cTAL content but different CCD content (groups 1 (n = 5) and 2 (n = 5)) or with similar CCD content but different cTAL content (groups 2 and 3 (n = 5)), and (**C**) subgroups with similar G content but different PCT content (groups 1(n = 6) and 2 (n = 4)) or with similar PCT content but different G content (groups 2 and 3 (n = 8)). Results indicate that **A**: DUSP9 and MUC1 were preferentially expressed in cTAL- and CCD-rich samples whereas GSTA1 and DCN were preferentially expressed in G- and PCT-rich samples. Statistical differences between groups: *, p<0.05; **, p<0.001; **B**: DUSP9 was preferentially expressed in cTAL whereas MUC1 was expressed in both cTAL and CCD because DUSP9 expression increased with cTAL content but not with CCD content, whereas MUC1 expression increased with both cTAL and CCD contents. Statistical comparison was performed between groups 1 and 2 and groups 2 and 3: *, p<0.01; **p<0.005; and **C**: GSTA1 and DCN were preferentially expressed in PCT and G respectively. Statistical comparison was performed between groups 1 and 2 and groups 2 and 3: **p<0.005. These conclusions are consistent with known expression profiles of these four genes (table 1).

One can take advantage of sample structural diversity to identify the site of expression of a gene, through selection of subgroups of samples with different compositions. Using this strategy, we found that DCN, GSTA1 and DUSP9 were preferentially expressed in G, PCT and cTAL respectively whereas MUC1 was expressed in both cTAL and CCD (**Fig. 6**). These conclusions are consistent with known expression profiles of these four genes (**Table 1**).

## Discussion

This paper describes a tissue compartment analysis method to quantify the proportion of the different cellular structures in a kidney tissue sample, such as a surgical piece of kidney or a renal biopsy. The method was initially designed and validated using normal kidney tissue because it is a readily available source of tissue, and because we disposed of published data regarding the segmental expression of thousands of genes in such tissue. However, TCA proved also efficient for analyzing kidney needle biopsies from patients with a wide variety of kidney diseases.

TCA revealed the extreme diversity of composition of kidney samples (Fig. 3). Consequently, use of a standardization factor that eliminates expression variability linked to differential sample composition and differential gene expression in the various structures proved to be highly efficient for reducing inter-sample data scatter (Fig. 4) and, thereby, for identifying pathological biomarkers (Fig. 5). Of course, the beneficial effect of standardization on data scattering mainly depends on how the considered gene is expressed in the various structures. For a gene evenly expressed in all compartments, standardization will have no effect, whereas the effect will be maximal for a gene expressed in a single compartment.

As demonstrated by examples illustrated in figure 6, the wide structural diversity of samples can be utilized to select sub-groups of samples with different enrichment in a given structure, and thereby to identify gene expression profile along the nephron. Interestingly, this approach can be developed at a single kidney level, since multiple sampling of a same kidney yields the same diversity as sampling from different kidneys (Fig. 3C).

Since the easy-to-use TCA method proved to be efficient in overcoming the kidney high heterogeneity, it could be applied successfully to any heterogeneous tissue inasmuch structure specific markers with defined expression levels are available. For example, TCA could be applied to central nervous system where tissue heterogeneity is a limiting factor [18], and specific structure markers are defined [19]. Choice of putative markers can be orientated by functional knowledge on tissues, and their quantification can be obtained indifferently by either RT-PCR or SAGE or microarray.

For application to kidney tissue, potential users should quantify by RT-qPCR their gene(s) of interest along with the 8 structure markers here proposed and the general marker RPLP1. In addition, they should look for gene specific tag abundance in GEO-deposited SAGE libraries. From these data, they can calculate Vs and SFs as indicated above and normalize data. For application to other heterogeneous tissues, potential users will first have to identify and validate couples of compartment-specific markers. For this purpose, the three criteria selected above (high expression level, responsible for compartment-specific function, and low inter-individual differences) might be helpful.

TCA is primarily well suited for human studies, because the small size of tissue samples collected in clinical practice is associated with high structural diversity. Its two main domains of application are: a) the follow up of identified biomarkers in tissue biopsies from single patients for diagnosis and/or prognosis as well as for evaluation of therapy efficiency, and b) the identification of new pathological markers through large scale analysis of tissue libraries. For this later application, it is worth pointing out that *a posteriori* analysis of existing data might be possible since specific probes or tags for structure markers are likely present on commercially available microarrays or in SAGE databases, respectively. TCA method may also be of interest in experimental studies for longitudinal follow up of single animals by repeated tissue biopsies. Spreading out TCA method to data analysis is expected to boost the outcomes of high throughput gene expression studies, especially to reveal otherwise imperceptible gene expression changes and for discovery of molecular markers of diseases.

## Methods

### Human Kidney Samples

Normal kidney tissue samples were obtained from 94 patients who had undergone surgery at the Department of Urology (North Hospital, CHU of Saint-Etienne, France) for kidney tumors. Written informed patient consent was obtained for studying gene expression profiles, and the study protocol was approved by the “Comité Consultatif de Protection des Personnes dans la Recherche Biomédicale Rhône-Alpes Loire”, France. After surgery, a kidney fragment taken at distance from the tumor, and later characterized as normal on histological basis, were snap-frozen with liquid nitrogen and stored at −80°C until studies. At the time of study, tissue samples were broken into smaller fragments in a mortar cooled in liquid nitrogen, and individual fragments were processed directly for RNA extraction.

Pathological kidney tissue was from the bio-library established by the Nephrology department at European Georges Pompidou Hospital (Paris, France), where needle biopsies are routinely collected at nephropathy diagnosis or during the follow up of the kidney disease. We retrospectively randomly selected 41 patients whose nephropathy was minimal change disease (n = 21), idiopathic membranous nephropathy (n = 7), diabetic glomerulosclerosis (n = 7), systemic erythematous lupus nephritis (n = 7), IgA nephropathy (n = 5), and renal sarcoidosis (n = 3).

### Preparation of Serial Tissue Sections

Frozen normal kidney tissue samples from two patients were stuck on tissue holders with tissue freezing medium. Three 8-µm serial frozen sections were cut on a standard cryostat with a clean blade. Two sections used for immunostaining were mounted on poly-L–lysine-precoated glass slides (Menzel-glaser, Germany), and successively dried at room temperature for 15 min, fixed for 2 min in ice-cold acetone, dried for 15 min, and rapidly washed with 1X phosphate-buffered saline (PBS), pH 7.4, before staining.

The 3^rd^ tissue section was used for laser capture microdissection (LCM) and RNA extraction. It was mounted on a slide coated with a thermoplastic membrane (PEN foil slides; Leica Microsystems, Wetzlar, Germany) and successively fixed with 70% ethanol at −20°C for 1 min to minimize nucleic acid degradation, thoroughly air dried, and stained for 45 sec at room temperature with a modified toluidine blue staining procedure. Thereafter, it was rapidly rinsed with xylene, and air dried before LCM. From each toluidine blue-stained section, four rectangular regions of approximately 1×1.4 mm were dissected with a Leica SVS LMD System (Leica Microsystems). The tissue section was photographed before and after LCM. Individual LCM-derived pieces of tissue were collected by gravity into a microcentrifuge tube containing 65 µl RLT buffer of the RNeasy Micro Kit from Qiagen complemented with β-mercaptoethanol for immediate RNA extraction.

### Immunostaining

The two remaining serial sections were used for immunostaining cTAL and CCD respectively. For CCD, acetone-fixed sections were incubated for 30 min at room temperature with the murine monoclonal anti-AQP2 antibody 1321 (1∶1000 dilution, Santa Cruz Biotechnology), rinsed thrice with PBS, and incubated with TRITC-conjugated goat anti-mouse immunoglobulin (1∶100 dilution, Sigma) for 30 min at room temperature. For cTAL staining, acetone-fixed sections were incubated with a human anti-uromucoid goat antibody (1∶200 dilution, Cappel) for 30 min at room temperature, rinsed thrice in PBS, and incubated with FITC-conjugated goat anti-rabbit immunoglobulin (1∶50 dilution; Dako Cytomation) for 30 min at room temperature. Before mounting for microscopic observation, slides were rinsed twice for 5 min in PBS.

### Histo-Morphometric Analysis of Serial Tissue Sections

Comparison of the micrographs of toluidine blue-stained sections, before and after LCM, with cognate immuno-stained sections allowed localizing the dissected zones on the three stained sections (Fig. 2A), and their histological composition was analyzed using Photoshop (Adobe Photoshop 6.0). After delineating cTALs and CCDs on the corresponding immuno-stained micrographs, Gs and PCTs were characterized on morphological basis on toluidine blue-stained sections (Fig. 2B) and delineated. Based on this histological analysis, a five color-coded image of each LCM-derived zone was constructed (Fig. 2C), and the overall surface area of each of the four structures of interest (G, PCT, cTAL and CCD) was determined (in pixels), expressed as percent of the sum of the four structures, and compared with data from TCA method (Fig. 2D). Note that this analysis does not take into account the fifth compartment (in grey in Fig. 2C) consisting in other nephron segments (mainly distal convoluted tubule), vascular structures and interstitial tissue. Histological and morphometric analysis of each LCM-derived zone was performed by two independent investigators and results are mean values of the two observations.

### RNA Extraction

RNAs from LCM-derived tissue pieces were immediately extracted using RNeasy Micro kit (Qiagen) according to the manufacturer's protocol for isolation of nucleic acids from microdissected cryosections. After DNase digestion (RNase-free DNase, Qiagen), the total RNA was washed and eluted with 14 µl of RNase-free water.

RNAs were also extracted from 10–20 mg frozen fragments of normal tissue and from entire biopsies of pathological tissue (2–3 mg), using RNeasy Mini and Micro Kit (Qiagen), respectively. Briefly, tissue fragments were homogenized with 350 µl of RLT complemented with β-mercaptoethanol in a spin/rotation instrument (FastPrep-120; Q BIOgene, 45 sec, speed 6). After centrifugation (10,000 g, 3 min at room temperature), the cell lysate (supernatant) was transferred onto spin-column (Qiagen) and treated according to the manufacturer's instructions for isolation of nucleic acids from animal tissues. After DNase digestion (RNase-free DNase, Qiagen), the total RNA was washed and eluted with RNase-free water (45 and 15 µl for normal and pathological tissue respectively).

RNA samples quality was checked on a Bioanalyzer 2100 System (Agilent Technologies, Palo Alto, CA, USA) and their concentration was confirmed by measuring their OD at 260 nm before reverse transcription.

### Reverse Transcription and Quantitative Real-Time Polymerase Chain Reaction (qPCR)

Reverse transcription was carried out in a final volume of 20 µl containing 10 U M-MLV reverse transcriptase (Roche), 1 mmol/l dNTPs (Eurobio, France), 20 U RNase inhibitor (Roche), 60 pmol/µl random hexamers (Roche) and approximately 200 ng RNA. Reverse transcription was carried out for 60 min at 42°C, followed by 5 min at 95°C and 5 min at 4°C.

PCR was carried out in 96-well plates using a LightCycler® 480 system (Roche Dagnostics). The reaction mixture contained 5 µl of LightCycler® 480 SYBR Green I Master mix (Roche Diagnostics), 0.5 µl of sense and antisense primers (0.5 µM final concentration) and 4 µl of reverse transcription product that was diluted so as to add a cDNA amount corresponding to 0.4 ng reverse-transcribed RNA. The reaction protocol included: 5 min at 95°C, followed by 45 cycles consisting of 10 sec at 95°C, 20 sec at 60°C and 20 sec at 72°C, and terminated with a melting curve analysis (from 60°C to 95°C) to check the specificity of the PCR product. Specific primers (**Table 2**) were designed using the Light Cycler probe design software.

**Table 2:** Sequence of nucleotide primers used for PCR.

Gene	Sense	Antisense
ALDOB	5′- gaggattgccgaccag-3′	5′- ggtcattcagggcctt-3′
AQP2	5′- cacgcattactagaatcattt-3′	5′- ggttcaaggtatgaccca-3′
SERPINH1	5′-ggtaccagccttggatact-3′	5′- gggcaggcagaatgacta-3′
COL4A5	5′- ggccctcacattcctccta-3′	5′- cctgaaataccagttccaatgc-3′
CTGF	5′- ctagagaagcagagccgc-3′	5′- agaatttagctcggtatgtcttca-3′
DCN	5′- caacacgcctcatctg-3′	5′- aagactcacacccgaata-3′
DUSP9	5′-gcatccgctacatcct-3′	5′-cagtgacggtgacaga-3′
FXYD4	5′- gccaataaagacgatccc-3′	5′- gggcgagtttaattcataaag-3′
GSTA1	5′- atcgctacttccctgc-3′	5′- tgactgcgttattaaaacct-3′
KCNJ1	5′- gtggtatgcagtagcg-3′	5′- agccactcggattagg-3′
MUC1	5′-gtcagcgtgagtgatgt-3′	5′-gtactcgctcataggatgg-3′
NPHS2	5′- atttgctaccgaatgg-3′	5′- gcaatcatccgcactt-3′
PODXL	5′- agaattgctactcgaagg-3′	5′- gctagtgaccgtgaca-3′
PPIA	5′-gcatacgggtcctggcatctt-3′	5′-acatgcttgccatccaaccac-3′
PTGER1	5′- gtcggtatcatggtggtgtc-3′	5′- ggatgtacacccaagggttc-3′
RPLP1	5′- cacggaggataagatcaat-3′	5′- gcccatgtcatcatcaga-3′
RPL19	5′-tgctcacaagataccgtg-3′	5′-agacaaagtgggaggtt-3′
SLC13A3	5′- gctgacatctcgaccc-3′	5′-aacatgcttaccacttaagg-3′
SLC12A1	5′- ggagacctgcgtatgg-3′	5′- tggtaaaggcgtgagt-3′
TRPC6	5′- catattcattatggtgtttgtggc-3′	5′- ctgatttcacttcagaaagtccaaatatag-3′
UMOD	5′- ccagaccccttcctac-3′	5′- cagcaaaccggaacat-3′
WT1	5′- ccaggccaggatgtttcctaa-3′	5′ctcatgcttgaatgagtggttg-3′

Specific primers were designed using the Light Cycler Probe Design software. Gene symbols are from HUGO.

The initial number of cDNA copies (W) was calculated as: 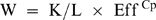
in which K is a constant, L is the amplicon length (in bp), Eff is the PCR efficiency and C_p_ is the number of PCR cycles where the reaction fluorescence reaches its second derivative maximum (threshold of fluorescence detection). Because K is unknown, this method does not allow the absolute quantification of a given transcript, but it permits calculating the relative abundance of two transcripts W_1_/W_2_.

Eff was determined using a standard curve made from 10-fold serial dilutions of a standard cDNA stock solution made from a mixture of 10 samples, and C_p_ was calculated by the LightCycler® 480 software. For each couple of primers, PCR was done twice for samples and four times for the standard curve, and mean Eff and Cp values were taken for calculations. Because Eff is raised to the Cp^th^ power, its determination must be as accurate as possible.
